# Childhood Physical and Sexual Abuse History and Leukocyte Telomere Length among Women in Middle Adulthood

**DOI:** 10.1371/journal.pone.0124493

**Published:** 2015-06-08

**Authors:** Susan M. Mason, Jennifer Prescott, Shelley S. Tworoger, Immaculata DeVivo, Janet W. Rich-Edwards

**Affiliations:** 1 Connors Center for Women’s Health and Gender Biology, Brigham and Women’s Hospital and Harvard Medical School, Boston, Massachusetts, United States of America; 2 Channing Division of Network Medicine, Department of Medicine, Brigham and Women’s Hospital and Harvard Medical School, Boston, Massachusetts, United States of America; 3 Program in Genetic Epidemiology and Statistical Genetics, Department of Epidemiology, Harvard School of Public Health, Boston, Massachusetts, United States of America; 4 Department of Epidemiology, Harvard School of Public Health, Boston, Massachusetts, United States of America; University of Newcastle, UNITED KINGDOM

## Abstract

**Objective:**

Abuse victimization in childhood is associated with a variety of age-related cardiometabolic diseases, but the mechanisms remain unknown. Telomeres, which form the protective caps at the ends of chromosomes, have been proposed as measures of biological age, and a growing body of research suggests that telomere attrition may help to explain relationships between stress and cardiometabolic degradation. We examined the association between childhood abuse victimization and leukocyte telomere length among 1,135 participants in the Nurses’ Health Study II (NHSII).

**Methods:**

The NHSII ascertained physical and sexual child abuse histories in 2001. Telomere length was measured in genomic DNA extracted from peripheral blood leukocytes collected between 1996 and 1999. The ratio of telomere repeat copy number to a single gene copy number (T/S) was determined by a modified version of the quantitative real-time PCR telomere assay. Telomere length was log-transformed and corrected for assay variation across batch. We regressed telomere length on childhood abuse exposure variables and covariates using linear regression.

**Results:**

We observed a reduction in telomere length associated with moderate physical abuse versus no physical abuse, but there was no evidence of a dose-response relationship for increased severity of physical abuse. No associations were noted for sexual abuse.

**Conclusions:**

We found no evidence of an association between severity of childhood physical or sexual abuse and leukocyte telomere length in the NHSII.

## Introduction

Child abuse is a highly prevalent early life stressor, with roughly 40% of American women reporting a history of physical abuse in childhood, and 30% reporting a history of childhood sexual abuse [1,2]. Emerging evidence links child abuse victimization to a variety of age-related diseases, including hypertension [3], type 2 diabetes [4,5], and cardiovascular events [6,7], but the mechanisms underlying these associations remain unclear. It has been suggested that chronic stress, such as that experienced in response to abuse victimization, may accelerate biological ageing [8].

Telomeres, which form protective caps at the ends of chromosomes, are dynamic structures composed of long hexameric repeats complexed to a core set of proteins [9]. Telomeric DNA is incompletely replicated with each cell division [10], causing telomeres to shorten over time in most somatic tissues [11]. Evidence suggests that exposure to oxidative stress further contributes to telomere loss [12]. Thus, telomere shortening may be a better indicator of biological degradation than chronological age alone [11,13]. A growing body of research suggests that telomere attrition may help to explain relationships between established cardiometabolic risk factors and disease outcomes; for example, age, smoking, excess calorie consumption, and inflammation have been associated with shorter mean leukocyte telomere length [11,14–21], which, in turn, has been associated with increased risk of atherosclerosis, myocardial infarction, insulin resistance, and type 2 diabetes [11]. Understanding the association of abuse victimization with telomere length may help clarify the mechanism linking abuse victimization to cardiometabolic disease.

To date, seven published studies have investigated child maltreatment associations with telomere length. Three reported an association between child maltreatment and shorter telomere length [22–24], with one [24] prospectively documenting accelerated change in telomere length in childhood subsequent to violence exposure. Four studies reported no association between childhood maltreatment and telomere length [25–28], although two of these [26,27] did report associations for other child adversities (e.g., parental alcohol abuse) and another [28] found an association when abuse was combined with childhood separation from parents. Other studies have found that being institutionalized in childhood [29], witnessing family violence [30], and exposure to a large number of childhood adversities [31] are associated with shorter telomere length, while another found that children at high risk for maltreatment had shorter telomere length than those at low risk [32]; however, none of these explicitly examined childhood abuse.

The quality of the current evidence on child maltreatment and telomere length is variously limited by small samples and blunt child abuse measures, with the largest studies [25–26,28] examining abuse as a dichotomous exposure without information on severity. Given substantial differences in the estimated health impacts of severe versus milder forms of abuse [6], adequately powered abuse–telomere length studies that include measures of abuse severity are needed to provide evidence for or against the abuse–telomere length hypothesis. We therefore assessed the associations between severity of childhood physical and sexual abuse and telomere length in a sample of 1,135 women from the Nurses’ Health Study II.

## Methods

### Sample

The Nurses’ Health Study II (NHSII) follows 116,430 female registered nurses recruited in 1989, when they were between the ages of 25 and 42. Biennial questionnaires are used to gather sociodemographic, behavioral, and medical data. In 2001, a supplemental questionnaire asking about experiences of physical and sexual abuse in childhood (the “Violence Questionnaire”) was sent to 91,297 NHSII participants who had responded to the previous biennial questionnaire within three mailings. Questionnaires were returned by 68,376 (75%) of the Violence Questionnaire recipients. Of these, 24,961 had a blood sample available. Blood collection was conducted between 1996 and 1999 and has been described previously [33]. Briefly, we shipped women the supplies for the blood collection, women arranged to have their blood drawn, and shipped the samples with an icepack, via overnight courier, to our laboratory where it was processed and separated into plasma, red blood cell, and white blood cell components.

The current study was a cross-sectional analysis of Violence Questionnaire responders with an available blood sample who were selected as controls for two case-control studies, one of type 2 diabetes (T2DM; n = 677 controls) and one of cardiovascular disease (CVD; n = 327 controls). Of the CVD controls, 17 also happened to be sampled as T2DM controls and thus had telomere length measured twice; we used the telomere length value that had the smallest within-sample coefficient of variation (described further in the Outcome section). To ensure adequate numbers of exposed women, the study population was supplemented with a sample of women who reported high levels of abuse victimization. Women in the high-violence sample were similar to women in the control sample on most covariates, except that they more frequently reported a family history of type 2 diabetes and cardiovascular disease (S1 Table). The final study sample included 986 T2DM and CVD controls plus 155 women reporting a high level of abuse victimization on the Violence Questionnaire and who were free of prevalent T2DM or CVD at the time of blood collection, making a total of 1,141 blood samples assayed for telomere length. Of these, 6 failed the assay, leaving 1,135 samples with complete telomere length data for analysis.

### Ethics Statement

Questionnaire return constitutes implicit consent for participation in the NHSII. For blood collection, participants were mailed a collection kit with a cover letter asking nurse-participants to collect and return blood samples and explaining use of the samples. Return of the samples constituted implicit consent for participation in studies using the samples. This study, including consent procedures, was approved by the Institutional Review Board of Brigham and Women’s Hospital.

### Variables and variable definitions

#### Exposures

In previous analyses in this cohort, we reported that physical and sexual abuse experienced in childhood or adolescence (up to and including age 17) were associated with hypertension [3], type 2 diabetes [4], and cardiovascular events [6]. Child/adolescent physical abuse was assessed using questions from the Revised Conflict Tactics Scale [34], which asked participants to report the frequency with which a parent, step-parent or adult guardian pushed, grabbed, or shoved; kicked, bit, or punched; hit with something that hurt; choked or burned; or physically attacked the participant when she was a child (age 0–10) or adolescent (age 11–17). As in previous analyses [3–4], we categorized child/adolescent physical abuse into four categories, using the most severe event a participant reported during childhood or adolescence: none, mild (being pushed, grabbed, or shoved at any frequency or being kicked, bitten, or punched once or hit with something once), moderate (being hit with something more than once or physically attacked once), and severe (being kicked, bitten, or punched or physically attacked more than once or ever choked or burned).

Child/adolescent sexual abuse was ascertained by asking participants whether, as a child (age 0–10) or adolescent (age 11–17), they had ever been touched in a sexual way by an adult or an older child or forced to touch an adult or an older child in a sexual way when they did not want to, and whether, as a child, an adult or older child had ever forced or attempted to force them into any sexual activity by “threatening you, holding you down, or hurting you in some way when you did not want to?”[35]. Participants were asked to indicate whether this never happened, happened once, or happened more than once. We categorized child/adolescent sexual abuse into three categories [6], using the most severe event reported in childhood or adolescence: none, sexual touching only, and forced sexual activity.

#### Outcome

Genomic DNA was extracted from peripheral blood leukocytes using the QIAmp (Qiagen) 96-spin blood protocol. PicoGreen DNA quantitation was conducted using a Molecular Devices 96-well spectrophotometer. Genomic DNA was subsequently dried down and resuspended to ensure accurate and uniform DNA concentrations. The ratio of telomere repeat copy number to a single gene copy number (T/S) was determined by a previously described modified version [36] of the quantitative real-time PCR telomere assay [37]. This PCR-based assay uses a high-throughput 384-well format of the Applied Biosystems 7900HT PCR System. Briefly, 5 ng of peripheral blood leukocytes-derived genomic DNA were dried down in a 384-well plate and resuspended in 10 μL of either the telomere or 36B4 (single copy gene) PCR reaction mixture. Triplicate reactions of each assay were done on each sample. Relative telomere length (RTL) is reported as the exponentiated T/S ratio. The coefficient of variation (CV) for the exponentiated T/S ratio of blinded quality control samples was 23% for T2DM cases and controls and 33% for CVD cases and controls, which were assayed with the violence-exposed control samples. Within-replicate CVs for participants and blinded QCs were much lower within plates, ranging from 10.7 to 19.4%. Further, when QC samples with within-triplicate CVs of greater than 20% were excluded, CVs dropped to 14–26%. Finally, non-blinded laboratory QCs were also included across plates, and the CVs in these samples averaged 11.5%. Overall, these results suggest that most of the variation occurred between batches.

We took the natural log of the RTL, which deviated significantly from normality (Shapiro-Wilk p-value<0.001), giving us a normally-distributed log-RTL outcome. To control for variation across laboratory batches, we used the batch-correction method proposed by Rosner et al. [38]. First, we regressed log-RTL on indicators for laboratory batch and potential predictors of telomere length that might vary by batch, including participant’s year of birth; father’s age at birth; and age, smoking status (never/past/current), and body-mass index (kg/m2, continuous) at time of blood draw. We calculated a correction parameter for each batch by subtracting the average of all the batch parameter estimates from the parameter estimate for each individual batch. For each observation assayed within a particular batch, we adjusted the log-RTL by subtracting that batch’s correction parameter, producing a batch-adjusted log-RTL (henceforth simply log-RTL).

#### Covariates

In our main analyses, we included the following potential confounders as covariates in adjusted models: age at blood collection, paternal age at participant’s birth, race (indicators for African American, Asian, Hispanic, and other, with non-Hispanic white as the referent), mother’s and father’s educational attainment when participant was an infant (indicators for <9 years, 9–11 years, 12 years, 13–15 years, with 16+ as the referent), indictors for mother in a professional occupation and father in a professional occupation, indicator for parental home ownership when participant was an infant, continuous self-reported childhood body size at age 5 (the participant could choose one of nine female figures ranging from very lean, a score of 1, to very obese, a score of 9, that best represented her body type at age five years [39]), parental history of diabetes prior to age 60, parental history of myocardial infarction or stroke before age 60, and parental lifetime history of depression. We did not include adult measures of body weight, lifestyle, or depression, as these are likely to be influenced by childhood abuse victimization [40–42] and thus on the causal pathway from abuse to telomere length.

#### Data Analysis

We used linear regression to model log-RTL as a function of abuse severity and covariates. For each of our abuse exposures, we ran an age-adjusted model and a model adjusted for the additional potential confounders described above. We first modeled physical abuse and sexual abuse separately. We then examined their joint impact by modeling physical abuse with and without any sexual abuse (no physical or sexual abuse (referent); sexual abuse but no physical abuse; and mild, moderate, and severe physical abuse with and without sexual abuse), and sexual abuse with and without any physical abuse (no sexual abuse or physical abuse (referent); physical abuse but no sexual abuse; and sexual touching and forced sex with and without physical abuse). Physical abuse analyses excluded women missing information on physical abuse (n = 5), leaving 1130 for physical abuse analyses. Sexual abuse analyses excluded women missing information on sexual abuse (n = 8), leaving 1127 for sexual abuse analyses. Analyses of the joint effect of physical and sexual abuse excluded women missing either physical or sexual abuse (n = 11), leaving 1124 women for joint physical and sexual abuse analyses.

We ran several supplemental analyses to assess the sensitivity of our results to potential biases in our data. First, we were concerned that the outcome misclassification indicated by our high overall CV would drive our effect estimates toward the null. We therefore re-ran our analyses limiting our sample to those women whose within-replicate CVs (that is, CVs of the triplicate assays run for each participant) were less than 20%. Second, our study sample relied largely on healthy controls selected as part of T2DM and CVD case-control studies, and therefore excluded most women who developed T2DM and CVD after blood collection (i.e., cases in our case-control studies), who may be more likely to have a history of abuse [4,6]. We therefore re-ran our analyses in our study sample plus randomly sampled T2DM and CVD cases to match the frequency of T2DM and CVD in 2001 Violence Questionnaire responders who had contributed a blood sample (the cohort from which cases and controls were drawn). Third, we had supplemented our sample with 155 women reporting violence exposure, to ensure adequate numbers of exposed women, and we wished to make sure that the inclusion of this “violence-exposed” subsample did not affect our results in unforeseen ways. We therefore re-ran our analyses excluding these 155 women. Fourth, we re-ran our models among white participants and among never-smokers. We restricted to whites, because telomere dynamics have been found to differ by race [43–46], and we had too few non-whites in our sample to run stratified analyses. We restricted to never-smokers, since smoking is an important predictor of telomere length that might overshadow the impact of other factors. Finally, we examined abuse–log-RTL associations stratified by age at blood draw, as the strength of association between childhood abuse exposure and RTL may diminish over time as additional health insults are accumulated. Analyses were run in SAS 9.2.

## Results

Among 1,135 women in our study, parental history of depression was strongly related to abuse severity, with maternal depression reported by 9.9% of those with no history of physical abuse and 19.6% of those with a history of severe physical abuse (Table 1). Maternal diabetes and CVD prior to age 60 were also associated with abuse severity, but paternal diabetes and CVD history were not. Childhood SES variables were not strongly associated with abuse severity.

**Table 1:** Distribution of relative telomere length (RTL) and covariates across categories of childhood physical and sexual abuse severity: Nurses’ Health Study II .

Covariates		Physical abuse				Sexual abuse		
		None	Mild	Moderate	Severe	None	Touch only	Forced sex
		N = 470	N = 184	N = 283	N = 193	N = 660	N = 248	N = 219
Log-RTL^a^: Mean (SD)		-0.7(0.3)	-0.7(0.4)	-0.8(0.3)	-0.7(0.4)	-0.7(0.3)	-0.7(0.3)	-0.7(0.3)
Continuous covariates: Mean (SD)		Mean (SD)	Mean (SD)	Mean (SD)	Mean (SD)	Mean (SD)	Mean (SD)	Mean (SD)
Age at blood draw (years)		45.6(4.2)	45.3(4.1)	45.5(4.1)	45.6(3.9)	45.5(4.1)	45.7(4.2)	45.4(4.0)
Paternal age at participant birth (years)		31.5(6.6)	30.7(6.1)	30.2(6.1)	30.0(6.5)	30.9(6.3)	30.8(6.4)	30.3(6.6)
Mother’s education (years)		12.3(2.0)	12.4(2.0)	12.1(2.0)	12.0(2.1)	12.3(1.9)	12.4(2.1)	11.8(2.2)
Father’s education (years)		12.4(2.5)	12.7(2.4)	11.9(2.6)	12.1(2.3)	12.4(2.4)	12.3(2.7)	11.9(2.5)
Body size at age 5 years^b^		2.5(1.2)	2.4(1.2)	2.6(1.2)	2.5(1.4)	2.5(1.3)	2.6(1.2)	2.6(1.3)
Categorical covariates: Column %		%	%	%	%	%	%	%
Physical abuse								
	None	—	—	—	—	49.1	38.1	22.3
	Mild	—	—	—	—	16.3	17.4	15.5
	Moderate	—	—	—	—	22.6	28.7	28.5
	Severe	—	—	—	—	12.0	15.8	33.7
Sexual abuse								
	None	69.4	58.5	52.8	40.4	—	—	—
	Touched only	20.0	23.1	25.2	20.8	—	—	—
	Forced sex	10.6	18.4	22.0	38.8	—	—	—
Race/ethnicity								
	African-American	0.9	0.6	1.7	0.5	0.9	1.6	0.5
	Latina	0.4	1.2	2.1	2.0	0.9	0.8	2.3
	Asian	0.8	0.0	0.0	1.1	0.6	0.4	0.5
	Caucasian	94.3	95.2	92.2	94.4	94.3	94.3	93.2
	Other	1.7	2.3	2.2	1.5	2.3	1.2	1.4
Mother in professional occupation		10.3	13.0	13.1	10.6	11.0	14.5	9.1
Father in professional occupation		27.4	29.8	22.3	21.5	26.4	26.3	22.0
Parents owned home		47.3	56.9	44.1	41.7	49.1	49.4	40.0
Mother diagnosed with diabetes <age 60 years		3.5	4.1	4.4	7.3	3.9	4.0	7.0
Father diagnosed with diabetes <age 60 years		6.1	6.2	7.1	7.5	6.5	5.2	9.1
Mother diagnosed with CVD <age 60 years		3.6	5.6	5.6	8.0	5.2	4.4	6.4
Father diagnosed with CVD <age 60 years		16.6	17.4	21.8	13.2	17.3	16.5	19.1
Mother lifetime history of depression		9.9	11.3	14.2	19.6	11.4	10.5	18.3
Father lifetime history of depression		5.1	5.2	7.1	11.4	5.9	6.9	9.1

Physical abuse was based on most severe event and defined as none (no report), mild (being pushed, grabbed, or shoved at any frequency or being kicked, bitten, or punched once or hit with something once), moderate (being hit with something more than once or physically attacked once), and severe (being kicked, bitten, or punched or physically attacked more than once or ever choked or burned). Sexual abuse was defined as none, sexual touching only, and forced sexual activity and was based on most severe report in childhood or adolescence.

^a^Natural log of the relative telomere length.

^b^Participants chose the image of a female figure that best approximated their body type at age 5, ranging from 1 (very lean) to 9 (obese).

We estimated correlations between established predictors of telomere length and both RTL and log-RTL in our study population to confirm that our telomere length measure was behaving as expected in our sample (Table 2). Similar to previous findings [11,14], advancing age, increasing body mass index, and smoking were negatively correlated with telomere length and paternal age was positively correlated with telomere length in our sample.

**Table 2:** Correlations between established telomere length predictors and measured relative telomere length in the study sample.

Variable	Correlation with RTL (p-value)	Correlation with ln-RTL (p-value)
Participant age at blood draw (years, continuous)	-0.064 (p = 0.03)	-0.065 (p = 0.03)
Paternal age at participant’s birth (years, continuous)	0.061 (p = 0.05)	0.065 (p = 0.04)
Participant body mass index (kg/m2, continuous)	-0.038 (p = 0.21)	-0.043 (p = 0.16)
Participant smoking status (never, past, current; ordinal)^a^	-0.058 (p = 0.05)	-0.058 (p = 0.05)

Pearson correlation coefficients used for all variables except ordinal smoking status.

^a^Spearman correlation coefficient used in calculation.

We observed modest and non-significant reductions in log-RTL associated with moderate physical abuse compared to no abuse (age-adjusted ΔlogRTL = -0.038; 95% CI: -0.087, 0.011), which were unchanged by covariate adjustment (Table 3). This reduction is equivalent to an increase in 5.3 years of age (ΔlogRTL for 1 year increase in age = -0.007; 95% CI:-.0120, -0.003), but there was no evidence of a dose-response relationship for increased severity of physical abuse. No associations were noted for sexual abuse.

**Table 3:** Estimated difference in relative telomere length (RTL) by severity of childhood physical and sexual abuse.

Exposure		N (mean logRTL)	Model				
			Age-adjusted		Covariate-adjusted^a^	
			ΔlogRTL	95% CI	ΔlogRTL	95% CI
Physical abuse						
	None	470 (-0.736)	0	—	0	—
	Mild	184 (-0.752)	-0.019	(-0.076, 0.037)	-0.019	(-0.076, 0.038)
	Moderate	283 (-0.773)	-0.038	(-0.087, 0.011)	-0.037	(-0.087, 0.012)
	Severe	193 (-0.722)	0.013	(-0.043, 0.068)	0.020	(-0.037, 0.077)
	*p for trend*		*0*.*77*		*0*.*94*	
Sexual abuse						
	None	660 (-0.744)	0	—	0	—
	Touch only	248 (-0.740)	0.006	(-0.043, 0.054)	0.007	(-0.042, 0.056)
	Forced sex	219 (-0.749)	-0.005	(-0.056, 0.046)	-0.003	(-0.055, 0.049)
	*p for trend*		*0*.*91*		*0*.*99*	

^a^Adjusted for age at blood draw, paternal age at participant’s birth, race, participant’s mother’s education, participant’s father’s education, participant’s mother in a professional occupation, participant’s father in a professional occupation, parental home ownership when participant was an infant, childhood somatogram score, parental history of diabetes prior to age 60, parental history of myocardial infarction or stroke before age 60, parental history of depression.

When considering potential joint effects of physical and sexual abuse, we found no statistically significant associations or dose-response relationships. Among women reporting no history of sexual abuse, those with mild, moderate, and severe physical abuse (compared to no physical abuse) had non- significantly shorter average log-RTLs, equivalent to increases in age of 8.3 years (95% CI: -2.1, 18.6), 6.4 years (95% CI: -2.7, 15.7), and 10.3 years (95% CI: -1.4, 22.0), respectively (Table 4). However, those with severe physical abuse in combination with sexual abuse had non-significantly *longer* average log-RTL equivalent to a *reduction* in age of 8.1 years (95% CI: -18.6, 2.3).

**Table 4:** Estimated difference in relative telomere length (RTL) by combinations of physical and sexual abuse in childhood.^a^

Physical abuse	Without sexual abuse			With sexual abuse^b^		
	N (mean log-RTL)	ΔlogRTL	95% CI	N (mean log-RTL)	ΔlogRTL	95% CI
None	324 (-0.717)	0	—	143 (-0.776)	-0.060	(-0.125, 0.004)
Mild	107 (-0.770)	-0.058	(-0.130, 0.015)	77 (-0.727)	-0.011	(-0.094, 0.071)
Moderate	149 (-0.765)	-0.045	(-0.110, 0.019)	133 (-0.779)	-0.065	(-0.133, 0.003)
Severe	79 (-0.787)	-0.072	(-0.154, 0.010)	112 (-0.673)	0.057	(-0.016, 0.130)
Sexual abuse	Without physical abuse			With physical abuse^c^		
	N (mean log-RTL)	ΔlogRTL	95% CI	N (mean log-RTL)	ΔlogRTL	95% CI
None	324 (-0.717)	0	—	335 (-0.772)	-0.056	(-0.107, -0.004)
Touch only	94 (-0.769)	-0.052	(-0.128, 0.024)	153 (-0.720)	0.001	(-0.065, 0.064)
Forced sex	49 (-0.791)	-0.074	(-0.175, 0.026)	169 (-0.738)	-0.019	(-0.083, 0.044)

^a^Adjusted for age at blood draw, paternal age at participant’s birth, race, participant’s mother’s education, participant’s father’s education, participant’s mother in a professional occupation, participant’s father in a professional occupation, parental home ownership when participant was an infant, childhood somatogram score, parental history of diabetes prior to age 60, parental history of myocardial infarction or stroke before age 60, parental history of depression.

^b^Including sexual touching or forced sex.

^c^Including mild, moderate, or severe.

We found some evidence of reductions in log-RTL associated with sexual touching and forced sex among women *without* physical abuse, but none of the estimates were statistically significant. Effect estimates for sexual abuse in combination with physical abuse were null (Table 4).

None of our sensitivity analyses resulted in important alterations to our effect estimates (S2 Table).

## Discussion

In this study of 1,135 women in the Nurses’ Health Study II, we observed little evidence of an association between a history of childhood physical or sexual abuse and telomere length among middle aged women. We found somewhat greater support for a relationship between physical abuse and telomere length than for sexual abuse, but the effect estimates were not statistically significant and there was no evidence of a dose-response relationship. We ran several sensitivity analyses, to determine the influence of outcome misclassification, sampling strategy, and cohort characteristics, but none of these yielded important changes to our results.

Childhood abuse has been strongly associated with a number of health behaviors and chronic disease outcomes in the NHSII cohort, and these outcomes—including smoking [42], hypertension [3], type 2 diabetes [4], and cardiovascular events [6]—have in turn been found to be associated with shorter telomere length [11]. We therefore hypothesized that childhood and adolescent abuse would be associated with shorter telomere length. We did not find evidence for this hypothesis; overall we did not observe any clear associations of abuse and relative telomere length in a study of women who were middle-aged (though still primarily premenopausal). Our results are similar to those from three other published studies [25–27]. These studies used aggregate measures of childhood abuse or lumped all childhood abuse together into a single exposure category. We had hypothesized that the lack of severity measures in these studies was the reason for the null findings, based on previous work in which we had reported severe abuse was much more strongly associated with chronic disease risk than mild abuse [3,4,6]. However, we were unable to demonstrate an association between abuse and telomere length, despite using comparatively nuanced exposure categories. Although we had detailed abuse data, we were limited by a lack of information on other early adversities, such as parental drug dependency and alcohol abuse, which have been observed to be associated with telomere length in two studies [26,27] that found no association with abuse itself. That said, our study was larger than the three published studies that have shown a positive association between childhood abuse and telomere length [22–24].

The reasons for the variation in published study findings regarding abuse and telomere length are not clear, but telomere dynamics are known to be complex, with relative telomere length reflecting the cumulative inputs of both telomere erosion and compensatory telomerase activity, each of which may have a unique association with abuse victimization. For example, in a recent study, Chen et al. found adverse childhood experiences to be unassociated with relative telomere length, but associated with telomerase activity, at least among adults with major depressive disorder [47]. Unfortunately, we did not have data on telomerase activity and therefore could not examine this question. Differences in cohort characteristics may also be important. The three studies showing an association between childhood maltreatment and telomere length all focused on relatively young cohorts, with one looking at telomere length in childhood and the others in young adulthood (mean age 29 [22] and 30 [23]). In contrast, the four studies finding no association were conducted in older cohorts (mean ages ranged from 49 to 70 [25–27,31]); our study is more similar to these older cohorts, as participants in our study were on average 45.5 years of age at the time of blood draw. At older ages, accumulation of many other factors influencing telomere length could obscure the effects of early maltreatment, although we saw little difference in abuse–RTL associations in younger versus older women in our cohort (see S2 Table).

The relatively high inter-assay coefficient of variation for the relative telomere length assay is a limitation of our study, as it indicates potential measurement error in the outcome. We used a batch correction method that allowed us to control for misclassification of the outcome across assay batches. This method has been used successfully in prior studies to deal with batch-to-batch assay variation and reduce CVs, allowing observation of significant results [48,49]. In our study, this method did not substantively change the overall CV for our RTL estimates, but is important to note that RTL was nonetheless correlated as expected with established telomere length predictors, providing reassurance that our outcome is a reasonable measure of RTL. Further, since the assay was run in triplicate, we were able to run a sensitivity analysis excluding samples that had a within triplicate CV>20%, suggesting poor assay reproducibility. The associations in this subset of women with good assay reproducibility were similar to our findings in the overall analytic sample, although the sample size was reduced by about 20%. This suggests that our null results are unlikely to be fully explained by outcome misclassification.

The strengths of our study include information about abuse type and severity and exposure classifications that have demonstrated important dose-response associations with major chronic disease outcomes. In addition, we were able to adjust for important potential confounders, including childhood socioeconomic status and family history of chronic disease. Finally, we ran several sensitivity analyses to address the potential influence of outcome misclassification and sampling strategy.

In summary, we were unable to replicate the findings of several published studies showing an association between childhood abuse history and telomere length. Future investigations should consider a broader range of early adversities and different populations (e.g., men, younger or older populations) and alternative measures of biological age.

## Supporting Information

S1 Table Characteristics of high-violence sample versus control samples from case-control studies. (DOCX)

S2 Table Covariate-adjusted abuse–telomere length associations: Sensitivity analysis results. (DOCX)
