# Supplementary material for: Childhood Physical and Sexual Abuse History and Leukocyte Telomere Length among Women in Middle Adulthood
Source: PLoS One. 2015 Jun 8;10(6):e0124493. doi: 10.1371/journal.pone.0124493 (PMC4459951; doi:10.1371/journal.pone.0124493)
Supplement: S1 Table — (DOCX) [file pone.0124493.s001.docx]

S1 Table. Characteristics of high-violence sample versus control samples from case-control studies.

| Variables |  | Case-control samples (n=981) | High-violence sample (n=154) |
| --- | --- | --- | --- |
| Log-RTL^a^: Mean (SD) |  | -0.7(0.3) | -0.8(0.4) |
| Continuous covariates: Mean (SD) |  |  |  |
| Age at blood draw (years) |  | 45.6(4.1) | 45.0(4.0) |
| Paternal age at participant birth (years) |  | 12.3(2.0) | 12.1(2.3) |
| Mother’s education (years) |  | 12.3(2.5) | 12.4(2.5) |
| Father’s education (years) |  | 2.5(1.2) | 2.6(1.3) |
| Body size at age 5 years^b^ |  | 30.8(6.3) | 30.5(6.9) |
| Categorical covariates: Column % |  |  |  |
| Race/ethnicity |  |  |  |
|  | African-American | 1.1 | 0.7 |
|  | Latina | 1.3 | 0.7 |
|  | Asian | 0.5 | 0.7 |
|  | Caucasian | 93.8 | 94.2 |
|  | Other | 2.0 | 1.2 |
| Mother in professional occupation |  | 11.5 | 11.2 |
| Father in professional occupation |  | 25.6 | 26.2 |
| Parents owned home |  | 48.6 | 38.5 |
| Mother diagnosed with diabetes <age 60 years |  | 4.3 | 6.6 |
| Father diagnosed with diabetes <age 60 years |  | 6.3 | 8.9 |
| Mother diagnosed with CVD <age 60 years |  | 4.5 | 9.7 |
| Father diagnosed with CVD <age 60 years |  | 16.7 | 22.5 |
| Mother lifetime history of depression |  | 12.1 | 15.7 |
| Father lifetime history of depression |  | 6.3 | 8.7 |

^a^Natural lot of the relative telomere length

^b^Participants chose the image of a female figure that best approximated their body type at age 5, ranging from 1 (very lean) to 9 (obese)
