# Supplementary material for: Childhood Physical and Sexual Abuse History and Leukocyte Telomere Length among Women in Middle Adulthood
Source: PLoS One. 2015 Jun 8;10(6):e0124493. doi: 10.1371/journal.pone.0124493 (PMC4459951; doi:10.1371/journal.pone.0124493)
Supplement: S2 Table — (DOCX) [file pone.0124493.s002.docx]

S2 Table. Covariate-adjusted abuse–telomere length associations: Sensitivity analysis results.

| Sensitivity analysis | Type of abuse | Level of severity | N (mean log-RTL) | ΔlogRTL^a^ | 95% CI |
| --- | --- | --- | --- | --- | --- |
| Restricted to women with coefficients of variation <20% |  |  |  |  |  |
|  | Physical abuse |  |  |  |  |
|  |  | None | 354 (-0.685) | 0 | -- |
|  |  | Mild | 137 (-0.694) | -0.004 | (-0.070, 0.062) |
|  |  | Moderate | 194 (-0.704) | -0.019 | (-0.078, 0.039) |
|  |  | Severe | 145 (-0.672) | 0.021 | (-0.045, 0.086) |
|  | Sexual abuse |  |  |  |  |
|  |  | None | 491 (-0.690) | 0 | -- |
|  |  | Touch only | 188 (-0.690) | 0.004 | (-0.052, 0.060) |
|  |  | Forced sex | 147 (-0.671) | 0.018 | (-0.045, 0.082) |
| Original (control) sample plus random sample of 85 type 2 diabetes, myocardial infarction, and stroke cases |  |  |  |  |  |
|  | Physical abuse |  |  |  |  |
|  |  | None | 504 (-0.736) | 0 | -- |
|  |  | Mild | 197 (-0.757) | -0.023 | (-0.077, 0.032) |
|  |  | Moderate | 311 (-0.771) | -0.035 | (-0.082, 0.012) |
|  |  | Severe | 202 (-0.717) | 0.025 | (-0.030, 0.080) |
|  | Sexual abuse |  |  |  |  |
|  |  | None | 707 (-0.743) | 0 | -- |
|  |  | Touch only | 268 (-0.748) | -0.004 | (-0.050, 0.042) |
|  |  | Forced sex | 236 (-0.743) | 0.000 | (-0.050, 0.049) |
|  |  |  |  |  |  |
| Excluding high-violence sample (n=154) | Physical abuse |  |  |  |  |
|  |  | None | 447 (-0.732) | 0 | -- |
|  |  | Mild | 172 (-0.750) | -0.019 | (-0.077, 0.039) |
|  |  | Moderate | 256 (-0.773) | -0.039 | (-0.090, 0.012) |
|  |  | Severe | 106 (-0.708) | 0.032 | (-0.038, 0.103) |
|  | Sexual abuse |  |  |  |  |
|  |  | None | 627 (-0.743) | 0 | -- |
|  |  | Touch only | 234 (-0.750) | -0.005 | (-0.054, 0.045) |
|  |  | Forced sex | 117 (-0.719) | 0.027 | (-0.039, 0.092) |
| Restricted to white participants |  |  |  |  |  |
|  | Physical abuse |  |  |  |  |
|  |  | None | 443 (-0.735) | 0 | -- |
|  |  | Mild | 176 (-0.745) | -0.015 | (-0.074, 0.043) |
|  |  | Moderate | 261 (-0.785) | -0.046 | (-0.098, 0.005) |
|  |  | Severe | 182 (-0.725) | 0.014 | (-0.045, 0.072) |
|  | Sexual abuse |  |  |  |  |
|  |  | None | 622 (-0.747) | 0 | -- |
|  |  | Touch only | 234 (-0.740) | 0.009 | (-0.041, 0.060) |
|  |  | Forced sex | 204 (-0.751) | -0.002 | (-0.056, 0.051) |
| Restricted to never-smokers |  |  |  |  |  |
|  | Physical abuse |  |  |  |  |
|  |  | None | 330 (-0.731) | 0 | -- |
|  |  | Mild | 109 (-0.718) | 0.012 | (-0.058, 0.082) |
|  |  | Moderate | 178 (-0.778) | -0.051 | (-0.111, 0.009) |
|  |  | Severe | 104 (-0.725) | 0.024 | (-0.049, 0.097) |
|  | Sexual abuse |  |  |  |  |
|  |  | None | 451 (-0.740) | 0 | -- |
|  |  | Touch only | 155 (-0.733) | 0.001 | (-0.059, 0.060) |
|  |  | Forced sex | 111 (-0.740) | 0.002 | (-0.066, 0.071) |
| Restricted to women aged ≤45 years at blood draw |  |  |  |  |  |
|  | Physical abuse |  | 198 (-0.706) | 0 | -- |
|  |  | None | 85 (-0.752) | -0.042 | (-0.130, 0.046) |
|  |  | Mild | 127 (-0.717) | 0.001 | (-0.077, 0.079) |
|  |  | Moderate | 77 (-0.640) | 0.060 | (-0.031, 0.152) |
|  |  | Severe |  |  |  |
|  | Sexual abuse |  |  |  |  |
|  |  | None | 287 (-0.713) | 0 | -- |
|  |  | Touch only | 108 (-0.685) | 0.047 | (-0.029, 0.123) |
|  |  | Forced sex | 90 (-0.701) | 0.022 | (-0.061, 0.105) |
| Restricted to aged >45 years at blood draw |  |  |  |  |  |
|  | Physical abuse |  |  |  |  |
|  |  | None | 272 (-0.757) | 0 | -- |
|  |  | Mild | 99 (-0.752) | 0.019 | (-0.056, 0.094) |
|  |  | Moderate | 156 (-0.819) | -0.062 | (-0.127, 0.002) |
|  |  | Severe | 116 (-0.777) | 0.009 | (-0.064, 0.081) |
|  | Sexual abuse |  |  |  |  |
|  |  | None | 373 (-0.769) | 0 | -- |
|  |  | Touch only | 140 (-0.782) | -0.008 | (-0.072, 0.056) |
|  |  | Forced sex | 129 (-0.782) | -0.001 | (-0.068, 0.066) |

^a^Adjusted for age at blood draw, paternal age at participant’s birth, race, participant’s mother’s education, participant’s father’s education, participant’s mother in a professional occupation, participant’s father in a professional occupation, parental home ownership when participant was an infant, childhood somatogram score, parental history of diabetes prior to age 60, parental history of myocardial infarction or stroke before age 60, parental history of depression.
